# Allergy Enhances Neurogenesis and Modulates Microglial Activation in the Hippocampus

**DOI:** 10.3389/fncel.2016.00169

**Published:** 2016-06-28

**Authors:** Barbara Klein, Heike Mrowetz, Josef Thalhamer, Sandra Scheiblhofer, Richard Weiss, Ludwig Aigner

**Affiliations:** ^1^Institute of Molecular Regenerative Medicine, Paracelsus Medical UniversitySalzburg, Austria; ^2^Spinal Cord Injury and Tissue Regeneration Center Salzburg (SCI-TReCS), Paracelsus Medical UniversitySalzburg, Austria; ^3^Division of Allergy and Immunology, Department of Molecular Biology, University of SalzburgSalzburg, Austria

**Keywords:** allergy, neurogenesis, microglia, systemic inflammation, hippocampus, T_H_2 polarization

## Abstract

Allergies and their characteristic T_H_2-polarized inflammatory reactions affect a substantial part of the population. Since there is increasing evidence that the immune system modulates plasticity and function of the central nervous system (CNS), we investigated the effects of allergic lung inflammation on the hippocampus—a region of cellular plasticity in the adult brain. The focus of the present study was on microglia, the resident immune cells of the CNS, and on hippocampal neurogenesis, i.e., the generation of new neurons. C57BL/6 mice were sensitized with a clinically relevant allergen derived from timothy grass pollen (Phl p 5). As expected, allergic sensitization induced high serum levels of allergen-specific immunoglobulins (IgG1 and IgE) and of T_H_2 cytokines (IL-5 and IL-13). Surprisingly, fewer Iba1^+^ microglia were found in the granular layer (GL) and subgranular zone (SGZ) of the hippocampal dentate gyrus and also the number of Iba1^+^MHCII^+^ cells was lower, indicating a reduced microglial surveillance and activation in the hippocampus of allergic mice. Neurogenesis was analyzed by labeling of proliferating cells with bromodeoxyuridine (BrdU) and determining their fate 4 weeks later, and by quantitative analysis of young immature neurons, i.e., cells expressing doublecortin (DCX). The number of DCX^+^ cells was clearly increased in the allergy animals. Moreover, there were more BrdU^+^ cells present in the hippocampus of allergic mice, and these newly born cells had differentiated into neurons as indicated by a higher number of BrdU^+^NeuN^+^ cells. In summary, allergy led to a reduced microglia presence and activity and to an elevated level of neurogenesis in the hippocampus. This effect was apparently specific to the hippocampus, as we did not observe these alterations in the subventricular zone (SVZ)/olfactory bulb (OB) system, also a region of high cellular plasticity and adult neurogenesis.

## Introduction

In comparison to the broad interest focusing on the influence of T_H_1 inflammatory parameters on the central nervous system (CNS; e.g., Cunningham et al., 2009; Henry et al., 2009; Jurgens and Johnson, 2012; Jurgens et al., 2012; Kahn et al., 2012; Kranjac et al., 2012; Valero et al., 2014), only a small number of studies deals with the effects of T_H_2 immunity on the brain (e.g., Tonelli et al., 2009; Sarlus et al., 2012, 2013). This is in striking contrast to the fact that chronic T_H_2-polarized immune reactions, which are a main characteristic of allergies, affect a substantial and increasing part of the population world-wide (Bieber, 2011; Fiocchi, 2011; Pawankar, 2011). The WAO estimates that 400 million people in the world suffer from allergic rhinitis and 300 million from asthma (Brozek et al., 2010; Pawankar, 2011).

Allergies are misguided responses of the immune system in which normally non-pathogenic stimuli, such as tree and grass pollen, dust mites, or animal dander, lead to immune responses characterized by the synthesis of allergen-specific IgE antibodies, the activation of T_H_2 immune cells and the production of the key T_H_2 cytokines IL-4, IL-5, and IL-13 (for a review, see Bloemen et al., 2007; Galli et al., 2008). At later stages of a persisting allergic immune response, also other T_H_ subsets, e.g., T_H_1 and T_H_17, may be activated leading to an increased production of pro-inflammatory cytokines, such as IFNγ and TNFα, or the T_H_17 cytokine IL-17 (reviewed in Holgate and Polosa, 2008).

There is increasing evidence that allergic reactions might influence immune status and functions of the CNS. In a rodent model of allergic rhinitis, reduced social interaction and anxiety-like behavior were observed, accompanied by the induction of a T_H_2-biased cytokine mRNA profile (IL-4, IL-5, IL-13) in the olfactory bulb (OB) and the prefrontal cortex (Tonelli et al., 2009). In another model of airway-induced allergy, the allergic reaction was associated with increased levels of the immunoglobulins IgG and IgE in CNS tissue, and with enhanced tau phosphorylation (Sarlus et al., 2012), a risk factor for the development of Alzheimer’s disease (AD). Chronic airway-induced allergy in mice modifies gene expression in the brain toward insulin resistance and inflammatory responses (Sarlus et al., 2013). In mice, in which a food allergy was induced shortly after weaning, reduced social behavior, increased self-grooming, reduced alternation in the T maze as well as decreased dopamine levels in the prefrontal cortex were observed (de Theije et al., 2014). Another study in juvenile mice, which were exposed to a long-term OVA-based asthma regime, showed impaired learning and memory in the Morris water maze, disturbed long-term potentiation in the hippocampal CA1 region and reduced cell proliferation in the hippocampal neurogenic niche (Guo et al., 2013).

There are only few findings suggesting that allergic reactions, like allergic rhinitis and asthma, affect cognitive functions in humans. Individuals suffering from seasonal allergic rhinitis, for example, perform worse in cognitive tests (Hartgerink-Lutgens et al., 2009). Moreover, there is a positive correlation between allergic rhinitis and mood disorders, such as anxiety and depression (reviewed in Sansone and Sansone, 2011). Similarly, children with asthma have higher rates of depression, behavioral disorders, and learning disabilities (Blackman and Gurka, 2007; Blackman and Conaway, 2012). There is also a correlation between allergies and epilepsy in children (Silverberg et al., 2014). While elderly asthma patients can profit from anti-asthmatic treatment, at least temporarily, with improved cognitive functions (Bozek et al., 2010), patients suffering from seasonal allergic rhinitis have a slower processing speed during attention tasks—also during symptom-free periods (Trikojat et al., 2015).

While there are indications that chronic systemic inflammation might contribute to neurodegenerative diseases (reviewed in Perry, 2010; Czirr and Wyss-Coray, 2012; Cunningham, 2013), the data about a possible influence of allergy on neurodegeneration is still conflicting. A longitudinal study in a population-based twin sample showed a positive association between a history of atopy and dementia (Eriksson et al., 2008). However, another study reported recently that AD patients who also suffered from allergies had an improved biomarker profile, closer resembling that of healthy subjects (i.e., higher Aβ_42_ levels in the cerebrospinal fluid), and had a better cognitive performance, which might indicate a beneficial effect of allergy on AD (Sarlus et al., 2015).

There is accumulating evidence that the immune system, e.g., via cytokines and chemokines, strongly modulates CNS functions like learning and memory, and also adult neurogenesis, the generation of new neurons in the adult CNS (reviewed in Yirmiya and Goshen, 2011). Thus, the aim of the present study was to investigate if an allergic reaction influences the hippocampus, specifically the dentate gyrus, which contains one of two classical neurogenic niches in the adult CNS and which is known for its central role in cognitive functions (reviewed in Marín-Burgin and Schinder, 2012; Bond et al., 2015). In comparison, we also analyzed the subventricular zone (SVZ), from which neuronal progenitors migrate via the rostral migratory stream to the OB to integrate into the neuronal networks, the second classical neurogenic niche (reviewed in Bond et al., 2015).

Based on these data, we hypothesized that a systemic allergic reaction affects neurogenesis and microglia in the hippocampus. Further, we expected that the effect of a T_H_2-polarized allergic response on microglial activation might differ from the well described reaction to a systemic LPS challenge.

## Materials and Methods

### Animals

Female C57BL/6 mice (aged 10–12 weeks) were purchased from Charles River Germany and afterwards kept under standard animal housing conditions with free access to food and water at the animal facility at the University of Salzburg, Austria. All experimental procedures were approved by the Austrian Ministry of Science and carried out in compliance with International Ethical guidelines.

### Allergy Induction

Recombinant Phl p 5.0101 (Phl p 5) was purchased from Biomay AG. The animals were divided into two groups: controls (*n* = 9) and allergy model (*n* = 10). The control group received all treatments using only the vehicle solution (phosphate-buffered saline, PBS). Animals of the allergy group were immunized intraperitoneally (i.p.) with 1 μg Phl p 5 adjuvanted with Al(OH)_3_ (Alu-Gel-S from Serva) in PBS (50% v/v, total volume: 200 μl) at weeks 1, 2, and 7. In week 11, starting 4 days before the perfusion (day 75), this group was challenged three times with a daily dose of 5 μg Phl p 5 in 40 μl PBS intranasally (i.n.; on days 71, 74 and 75). During this procedure, all mice (also the controls) were briefly anesthetized with isoflurane.

### Analysis of Blood Parameters

Blood samples were taken at the end of the experiment (day 75), and incubated for 1 h at 37°C. After centrifugation (10 min), the sera were collected and stored at −80°C until measurements. Serum levels of Phl p 5-specific IgG1 and IgG2c were determined by a luminescence-based ELISA, and biologically functional IgE was measured *in vitro* by a rat basophil leukemia (RBL) cell assay. Additionally, cytokines, chemokines and the growth factor VEGFα were measured with a Luminex Multiplex Assay (Milliplex MAP Mouse Cytokine/Chemokine Magnetic Bead Panel, Merck) according to the manufacturer’s instructions.

### Luminescence-Based ELISA Assay to Analyze Serological IgG Levels

Levels of Phl p 5-specific IgG1 and IgG2c were determined using a luminescence-based ELISA assay as previously described (Weinberger et al., 2013). In short, 96-well plates for immunoassays (Greiner) were coated for 24 h at 4°C with recombinant Phl p 5 (per well 50 μl of 1 μg/ml Phl p 5 in PBS). Afterwards, plates were washed with 0.1% Tween-20 in PBS (v/v) and incubated with blocking buffer (0.1% (v/v) Tween 20 and 2% (w/v) skim milk in PBS, pH 7.5) for 1 h at RT, before washing the plates again. Then, the plates were incubated with serum diluted (1:10,000) in blocking buffer for 1 h at RT, washed again, before the horse radish peroxidase (HRP)-conjugated antibodies for the detection of IgG1 (Zymed) or IgG2c (Zymed; diluted 1:1000 in blocking buffer) were added to the wells for 1 h at RT. After that, the luminometric assay (BM chemiluminescence substrate, Roche) was developed by adding the substrate (luminol diluted 1:2 in H_2_O) to each well. After 3 min incubation, chemiluminescence (photon counts/s) was determined using an Infinite M200 Pro Plate Reader (Tecan).

### RBL Cell Assay to Measure Biologically Functional IgE

The serum level of IgE was measured using a RBL cell assay as previously described (Weinberger et al., 2013). Briefly, RBL-2H3 cells (ATCC CRL-2256) were seeded in 96-well culture plates (Greiner) at a density of 6 × 10^5^ cells/ml and grown over night in 100 μl culture medium per well at standard culture conditions (37°C, 95% relative humidity, 5% CO_2_). The culture medium was RPMI 1640 supplemented with 10% (v/v) heat-inactivated fetal calf serum, 100 U/ml penicillin and 100 μg/ml streptomycin, 4 mM L-glutamine, 2 mM sodium pyruvate, 10 mM HEPES, and 100 μM 2-mercaptoethanol. Next day, cells were incubated for 2 h with different serum dilutions (1:50, 1:100, and 1:200). Untreated wells were used to assess background and maximum release values. To remove unbound antibodies, plates were washed twice with 200 μl Tyrode’s buffer (137 mM NaCl, 2.7 mM KCl, 0.5 mM MgCl_2_, 1.8 mM CaCl_2_, 0.4 mM NaH_2_PO_4_, 5.6 mM D-glucose, 12 mM NaHCO_3_, 10 mM HEPES, and 0.1% (w/v) bovine serum albumin (BSA); pH 7.2). Then the cells were incubated for 30 min in 100 μl of 0.1 μg/ml recombinant Phl p 5 diluted in Tyrode’s buffer to induce crosslinking of FcεR-bound IgE and degranulation of RBL cells. To determine maximum release, cell membranes were disrupted by adding 10 μl of a 10% (v/v) Triton X-100 solution. After that, 50 μl of the cell culture supernatants were transferred into fresh 96-well plates (Greiner), where they were incubated for 1 h with 50 μl assay solution at a final concentration of 80 μM 4-methylumbelliferyl N-acetyl-b-D-glucosaminide (4-MUG, Sigma) in 0.1 M citrate buffer (pH 4.5). To stop the reaction, 100 μl glycine buffer (0.2 M glycine and 0.2 M NaCl, pH 10.7) were added and fluorescence (in relative fluorescence units) was measured in a fluorescence microplate reader (Infinite M200 Pro, Tecan). Background values were subtracted from all measured values, and the results were presented as percentage of the maximum release value.

### Detection of Proliferating Cells to Determine Cell Fate

For the detection of proliferating cells, a solution of 10 mg/ml bromodeoxyuridin (BrdU; Sigma-Aldrich) in 0.9% NaCl (w/v), in a dosage of 50 mg/kg body weight, was once injected i.p. in week 7 on day 47 (4 weeks before the end of the experiment).

### Bronchoalveolar Lavage (BAL) and Tissue Processing

In week 11, mice were deeply anaesthetized by i.p. injection of a mixture of ketamine (273 mg/kg body weight), xylazine (71 mg/kg body weight) and acepromazine (4 mg/kg body weight) in a physiological NaCl solution. During deep anesthesia (which was carefully evaluated), tracheotomy and a bronchoalveolar lavage (BAL) were performed. In short, the lungs were washed twice with 1 ml of ice cold PBS and the fluid was collected back into the syringe. This BAL fluid was stored on ice until flow cytometric analysis (FACS Canto II, BD Bioscience).

After BAL, mice were transcardially perfused, first with a 0.9% NaCl (w/v) solution, and then with phosphate-buffered 4% paraformaldehyde (pH 7.4). Afterwards, brains were removed, postfixed overnight in 4% paraformaldehyde, cryoprotected in phosphate-buffered 30% sucrose (w/v), and sectioned on dry ice with a sliding microtome. The sections (thickness: 40 μm) were stored at −20°C in a cryoprotection solution (made of equal parts glycerin, 0.2 M phosphate buffer, ethylene glycol and H_2_O).

### Analysis of the BAL Fluid

BAL samples were centrifuged (7 min, 250 × g, 4°C) and 450 μl of supernatants were taken and mixed with 50 μl of 10% BSA and 1% NaN_3_ in H_2_O and frozen at −80°C. Cytokine levels in the BAL fluid were analyzed using a Luminex Multiplex Assay (MILLIPLEX MAP Mouse Cytokine/Chemokine Magnetic Bead Panel, Merck) according to the manufacturer’s instructions.

For analysis of different immune cell populations in the BAL fluid, cell pellets were re-suspended in 100 μl of the remaining supernatant and transferred into a 96-well V-bottom plate (Greiner), and then centrifuged (5 min, 250 × g, 4°C). After that, cell pellets were re-suspended in 30 μl antibody staining mix: CD45-PerCP/Cy5.5 (1:400, 30-F11, Biolegend), CD4-BV421 (1:200, GK1.5, Biolegend), CD19-PE-Cy7 (1:100, 6D5, Biolegend), Gr-1-APC (1:200, RB6-8C5, eBioscience), Siglec-F-PE (1:200, E50-2440, BD Biosciences), CD8-FITC (1:100, 53-6.7, eBioscience) and incubated for 10 min on ice. Afterwards, the cells were washed with 100 μl FACS buffer (0.5% BSA and 2 mM EDTA in PBS), and then incubated for 5 min at RT in 100 μl Red Blood Cell (RBC) Lysis Buffer (eBioscience) to remove residual RBCs. After another washing step (using FACS buffer), cell pellets were re-suspended in 120 μl FACS buffer and transferred into FACS tubes. Cells were analyzed on a FACS Canto II flow cytometer (BD Bioscience) and data were recorded for 30 s at a rate of 120 μl/min to calculate the absolute cell numbers per BAL. For flow cytometric analysis, at first total leukocytes were gated based on their expression of CD45. The FSC/SSC plots were used to exclude cell debris, and then in FSC-W/FSC-A plots the single cells were gated. Neutrophils were identified based on their high Gr1 expression. The other cells were gated according to their expression of CD4, CD8, and SiglecF. SiglecF is highly expressed on eosinophils and monocytes. Monocytes were then separated from eosinophils by their autofluorescence in the BV-510 channel. In general, the observed CD19 staining was weak; therefore, CD19^+^ cells were identified after exclusion of the previously gated cell types.

### Immunohistochemistry

Immunohistological stainings were performed as previously described (Kandasamy et al., 2014), using the following antibodies and dilutions. Primary antibodies: rat anti-BrdU (1:500, BU1/75, AbD Serotec), rabbit anti-CD68 (1:500, ab125212, Abcam), rabbit anti-doublecortin (1:250, 4604, Cell Signaling), guinea pig anti-GFAP (1:500, GP52, Progen), rabbit anti-Iba1 (1:300, 019-19741, Wako), goat anti-Iba1 (1:250, ab107159, Abcam), anti-mouse MHCII (I-A/I-E; 1:100, 14-5321-82, eBioscience), mouse anti-NeuN (1:500, A60, Merck Millipore), mouse anti-PCNA (1:500, sc-56, Santa Cruz). Secondary antibodies: donkey anti-rat Alexa 488, donkey anti-goat, -mouse Alexa 568, donkey anti-rabbit, -guinea pig Alexa 647 (all 1:1000, Invitrogen, Life technologies), donkey anti-rat Cy5, donkey anti-mouse biotinylated (1:1000, Jackson Immuno Research), goat anti-rabbit biotinylated, rabbit anti-rat biotinylated (all 1:500, Vector Labs). Cell nuclei were stained with 4′,6-diamidino-2-phenylindole dihydrochloride at a concentration of 0.5 μg/μl (DAPI; Sigma-Aldrich).

Image documentation and analysis were done using a Zeiss Axioplan light microscope or a confocal scanning laser microscope (Zeiss LSM 700) with LSM Software (ZEN 2012) for fluorescent stainings.

### Quantitative Analysis of Immunohistological Stainings

Image acquisition and quantification were done blinded (i.e., without knowing group or mouse number). For quantitative analysis, a representative tenth of one brain hemisphere was analyzed by collecting every 10th section, with an interval of 400 μm between sections. This 10th of a hemisphere was used for immunohistochemistry with a chromogenic dye. The total number of stained cells within the regions of interest was counted using a Zeiss Axioplan light microscope. In the dorsal hippocampal dentate gyrus, the total numbers of PCNA^+^, BrdU^+^, Iba1^+^ and DCX^+^ cells were counted. In addition, the total number of BrdU^+^ cells in the SVZ was determined.

To investigate the cell fate of BrdU^+^ cells in the dorsal dentate gyrus, a BrdU/NeuN/GFAP fluorescence staining was analyzed. For each animal, z-stacks of the dorsal dentate gyrus were made in a 10th brain hemisphere on a Zeiss LSM 700 laser scanning microscope. In these image stacks all BrdU^+^ cells were counted, and at the same time, it was also determined if these cells were co-labeled for NeuN or GFAP. Thus, the percentage of BrdU^+^NeuN^+^ or BrdU^+^GFAP^+^ cells was determined per animal. Similarly, BrdU^+^ cells in the granular cell layer of the OB were analyzed in a region of 400 × 400 × 40 μm.

To analyze the activation state of Iba1^+^ cells in the dorsal dentate gyrus, the numbers of Iba1^+^MHCII^+^ and Iba1^+^CD68^+^ cells were counted in z-stacks (generated using a with Zeiss LSM 700 equipped with the Zeiss ZEN 2012 software) of four visual fields (400 × 400 × 40 μm) per animal. For the OB, one visual field of the granular cell layer was analyzed.

To estimate the analyzed volume for each region, the corresponding tissue area in the middle of the stack was measured and then multiplied by 40 μm. Then the cell densities were determined by dividing the total number of counted cells by this volume and presented as cells/mm^3^.

### Statistics

Data are shown as Mean + standard deviation (SD). Statistical significance was determined in Prism 5 (Graphpad Software Inc) using independent samples *t*-tests (the corresponding *p*-values are represented as: *p < 0.05, **p < 0.01, ***p < 0.001). To adjust for multiple comparisons between the two groups, we computed for each large family of comparisons (cytokines in serum, cytokines in BAL fluid, and cell types in BAL fluid) a Holm-Šídák correction for multiple *t*-tests (at a global alpha level of 0.05) and reported the multiplicity adjusted *p*-values (in brackets) in addition to the unadjusted *p*-values. Statistical outliers were identified using the Grubb’s test (*p* < 0.05) in the QuickCals GraphPad Software^1^.

## Results

### Allergic Mice Have a T_H_2-polarized Immune Reaction

**Figure 1:**
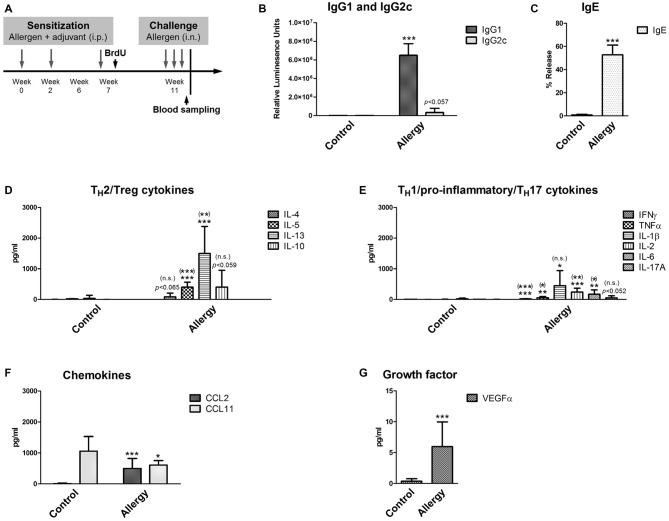
**Experimental design and serological analysis. (A)** Setup of the experiment. **(B–D)** Serological changes in Phl p 5-sensitized and rechallenged mice confirmed a T_H_2-polarized immune response. At the end of the experiment, sera were analyzed for **(B)** Phl p 5-specific IgG1 and IgG2c, and **(C)** biologically functional IgE. **(D)** Serum levels of T_H_2 cytokines (IL-4, IL-5 and IL-13) and the T_H_2/Treg cytokine IL-10. **(E)** Serum levels of T_H_1/pro-inflammatory cytokines (IFNγ, TNFα, IL-1β, IL-2, IL-6) and the T_H_17 cytokine IL-17A. **(F)** Levels of the chemokines CCL2 and CCL11 and of **(G)** the growth factor VEGFα in the serum. Data are shown as Mean + SD. (control: *n* = 9, allergy: *n* = 10). Statistical significance was determined using independent samples *t*-tests (*p < 0.05, **p < 0.01, ***p < 0.001; n.s. - not significant), multiplicity adjusted *p*-values (Holm-Šídák correction for multiple *t*-tests) are reported in brackets ((*)*p* < 0.05, (**)*p* < 0.01, (***)*p* < 0.001; (n.s.) - not significant).

The allergen used in the present study, Phl p 5, is derived from timothy grass pollen and frequently responsible for allergy symptoms in human patients (Matthiesen and Løwenstein, 1991; Sekerkova et al., 2012). After sensitization, lung inflammation was induced in C57BL/6 mice via intranasal application of the allergen (experimental set-up see Figure 1A). To exclude that any of the observed effects were caused by experimental procedures (e.g., handling of the animals or anesthesia), the controls underwent all experimental steps at the same time as the allergy group and received the vehicle solution (PBS) during sensitization and challenge.

First, the allergic status of the sensitized mice was confirmed by measuring blood and lung parameters. For this, the levels of allergen-specific immunoglobulins were determined. In mice, the T_H_2 cytokine IL-4 is necessary for the immunoglobulin class switch to IgG1 and IgE, whereas the T_H_1 cytokine IFNγ would cause a class switch to IgG2c. Thus, the immunoglobulin measurements (Figures 1B,C) showed that mice sensitized to the allergen had a T_H_2-polarized immune response, since we observed high IgG1 (Figure 1B) and IgE (Figure 1C) levels, whereas IgG2c was not significantly elevated in comparison to controls (Figure 1B).

As expected, high serum levels of T_H_2 cytokines IL-5 and IL-13 were observed in the allergy model (Figure 1D). The T_H_2 cytokine IL-4 was also modestly, but not significantly, elevated in the allergy model in comparison to controls. The cytokine IL-10 (Figure 1D), which can be derived from T_H_2 but also from regulatory Tr1 cells, was not significantly increased in allergic mice. Additionally, we found a modest increase of the pro-inflammatory cytokines IFNγ, TNFα, IL-1β, IL-2 and IL-6 in the sera of allergic mice (Figure 1E). Also, the serum levels of the T_H_17 cytokine IL-17A were slightly, but not significantly, elevated in the allergy model (Figure 1E).

The chemokine CCL2, which is an effective attractant for monocytes, was significantly increased in the allergy model, whereas surprisingly CCL11, a signaling molecule attracting eosinophils was significantly decreased in the serum of allergic mice (Figure 1F). The growth factor VEGFα was also increased in the allergy model (Figure 1G).

**Figure 2:**
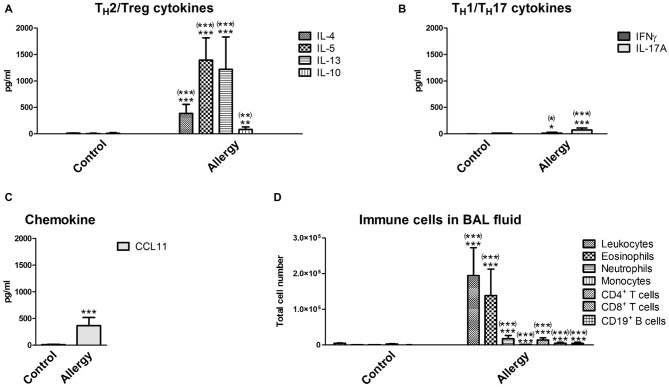
**Cytokines and immune cells in bronchoalveolar lavage (BAL) fluid.** Analysis of immune parameters in the BAL fluid confirmed a T_H_2-polarized immune reaction in the lungs of allergic mice.** (A)** BAL fluid levels of T_H_2 cytokines (IL-4, IL-5, and IL-13) and the T_H_2/Treg cytokine IL-10. **(B)** BAL fluid levels of the T_H_1 cytokine IFNγ, the T_H_17 cytokine IL-17A, and **(C)** the chemokine CCL11. **(D)** Immune cells which infiltrated into the lungs were analyzed in the BAL fluid using flow cytometry. Leukocytes, eosinophils, neutrophils, monocytes, T cells (CD4^+^ or CD8^+^) and CD19^+^ B cells were detected. Data are shown as Mean + SD. (control: *n* = 9, allergy: *n* = 10). Statistical significance was determined using independent samples *t*-tests (*p < 0.05, **p < 0.01, ***p < 0.001; n.s. - not significant), multiplicity adjusted *p*-values (Holm-Šídák correction for multiple *t*-tests) are reported in brackets ((*)*p* < 0.05, (**)*p* < 0.01, (***)*p* < 0.001; n.s. - not significant).

In the lungs of the allergic mice similar changes were observed. In the BAL fluid, the levels of the typical T_H_2 cytokines (IL-4, IL-5 and IL-13) and of IL-10 were increased (Figure 2A). The T_H_1 cytokine IFNγ and the T_H_17 cytokine IL-17A were also slightly increased (Figure 2B). In contrast to the serum, the chemokine CCL11 was markedly elevated in the BAL fluid, which was expected since CCL11 is important for the recruitment of eosinophils (Figure 2C). Indeed, the number of infiltrating leukocytes and eosinophils was very high in the BAL fluid of allergic mice (Figure 2D). Also neutrophils, T cells (CD4^+^ and CD8^+^) and CD19^+^ B cells invaded the lungs of the allergy model (Figure 2D). Unexpectedly, we found a small, but significant, reduction in the number of monocytes in the BAL fluid of allergic mice (Figure 2D).

### Allergy Modulates Microglia in the Hippocampal Neurogenic Niche

**Figure 3:**
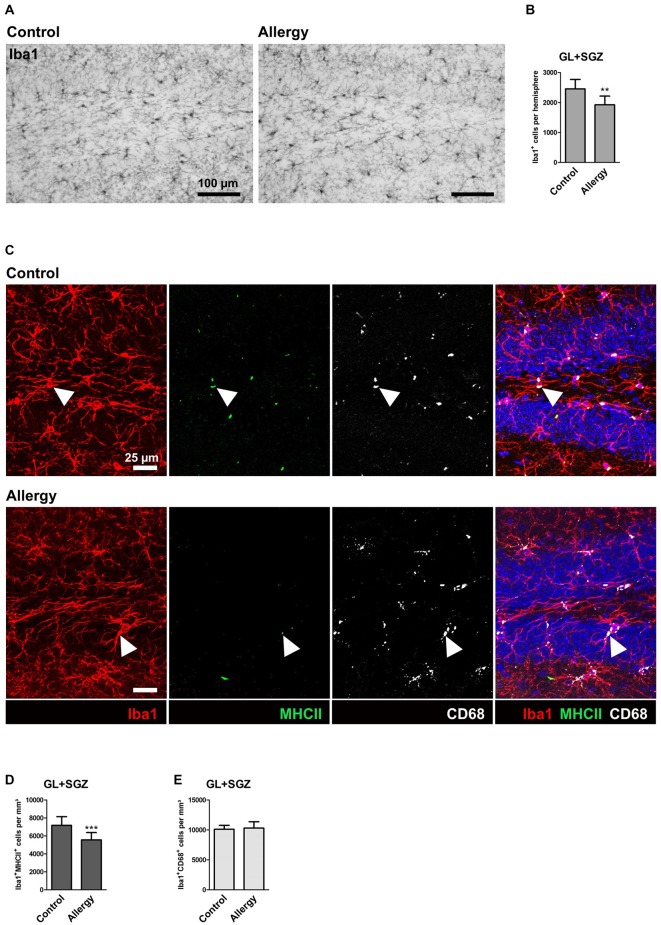
**Reduced numbers of Iba1^+^ microglia and of microglia expressing MHCII in the hippocampal neurogenic niche of allergic mice. (A)** Iba1^+^ microglia in the hippocampal dentate gyrus. **(B)** The number of Iba1^+^ cells in the granular layer (GL) and subgranular zone (SGZ) of the dentate gyrus is lower in allergic mice than in controls **(C)** Triple labeling of Iba1 (red), MHCII (green) and CD68 (white). Cell nuclei are stained with DAPI (blue). Triple-positive cells are indicated by arrow heads. **(D)** The number of Iba1^+^MHCII^+^ cells was decreased in the neurogenic niche of allergic mice, whereas **(E)** Iba1^+^CD68^+^ cells were not affected. Values are depicted as Mean + SD. (control: *n* = 9, allergy: *n* = 10). Statistical significance was evaluated using independent samples *t*-tests and is indicated in comparison to the control group (*p < 0.05, **p < 0.01, ***p < 0.001). Scale bars: **(A)** 100 μm, **(C)** 25 μm.

The neurogenic niche of the hippocampus is located in the subgranular zone (SGZ) of the dentate gyrus. The neurons which are generated from the neural stem cells in the SGZ then integrate into the granular layer (GL) of the dentate gyrus (reviewed in Bond et al., 2015). Microglia, the tissue macrophages of the CNS, play an important part in regulating the neurogenic niche (reviewed in Kokaia et al., 2012; Sierra et al., 2014). Since these cells are especially reactive to immune signals from the periphery (Hoogland et al., 2015), we checked whether they are influenced by allergy using the marker Iba1 which labels microglia and macrophages (Figure 3A). Surprisingly, a significant reduction in the number of Iba1^+^ cells was observed in allergic mice in the GL and SGZ of the dorsal hippocampal dentate gyrus (control: 2456 ± 295 cells per hemisphere, allergy: 1924 ± 275 cells per hemisphere; *p* < 0.0013; Figure 3B).

To check if also other commonly used markers for microglial activation were altered in allergic mice, we quantified MHCII, which is important for antigen presentation, and CD68, which is associated with lysosomes. In both, controls and the allergy group, these markers were mainly found in intracellular compartments, presumably lysosomes or endosomes (Figure 3C). While significantly fewer Iba1^+^ cells also expressed MHCII in allergic mice (control: 7172 ± 913 cells/mm^3^, allergy: 5555 ± 766 cells/mm^3^; *p* < 0.0001; Figure 3D), there was no change in the numbers of Iba1^+^CD68^+^ cells (control: 10103 ± 609 cells/mm^3^, allergy: 10300 ± 1002 cells/mm^3^; *p* < 0.6345; Figure 3E).

### Allergy Activates Microglia in the Granular Cell Layer of the OB

**Figure 4:**
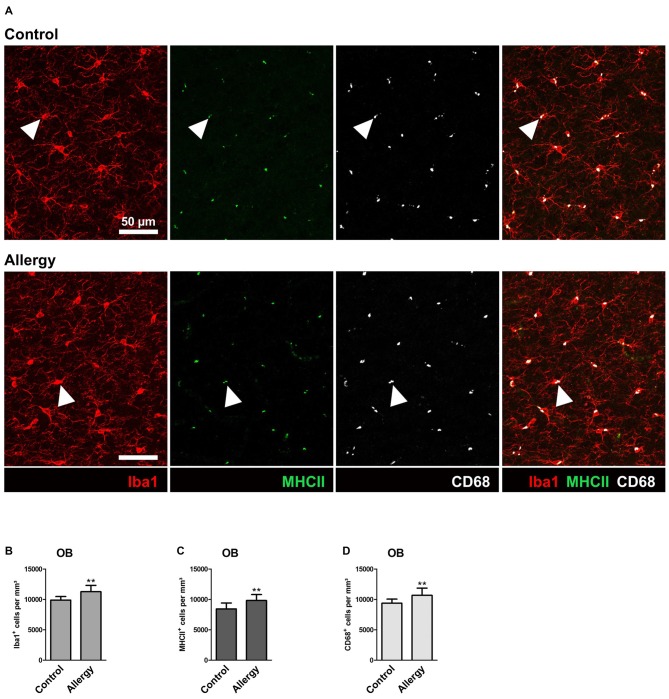
**Number of Iba1^+^MHCII^+^ and Iba1^+^CD68^+^ microglia is increased in the olfactory bulb (OB) of allergic mice. (A)** Triple labeling of Iba1 (red), MHCII (green) and CD68 (white). Triple-positive cells are indicated by arrow heads. **(B,C)** Increased numbers of **(B)** Iba1^+^, **(C)** Iba1^+^MHCII^+^ and **(D)** Iba1^+^CD68^+^ cells in the granular cell layer of the OB. Values are depicted as Mean + SD. (control: *n* = 9, allergy: *n* = 10). Statistical significance was evaluated using independent samples *t*-tests and is indicated in comparison to the control group (*p < 0.05, **p < 0.01, ***p < 0.001). Scale bars: 50 μm.

The OB is the brain region into which SVZ-derived newly generated neurons integrate (reviewed in Bond et al., 2015). Using the same markers as in the hippocampus, we analyzed microglial activation in the OB (Figure 4A) to find out if the changes we observed were specific for the hippocampus.

In contrast to the hippocampal neurogenic niche, allergy elevated the number of Iba1^+^ microglia in the granular cell layer of the OB (control: 9890 ± 546 cells/mm^3^, allergy: 11271 ± 985 cells/mm^3^; *p* < 0.0027) (Figure 4B). In parallel, also the numbers of Iba1^+^MHCII^+^ cells (control: 8433 ± 919 cells/mm^3^, allergy: 9829 ± 915 cells/mm^3^; *p* < 0.0060) (Figure 4C) and of Iba1^+^CD68^+^ cells (control: 9370 ± 638 cells/mm^3^, allergy 10676 ± 1123 cells/mm^3^; *p* < 0.0098) (Figure 4D) increased. This indicates that allergy leads to more activated microglia in the OB.

### Allergy Increases the Number of Immature DCX^+^ Neurons in the Hippocampus

**Figure 5:**
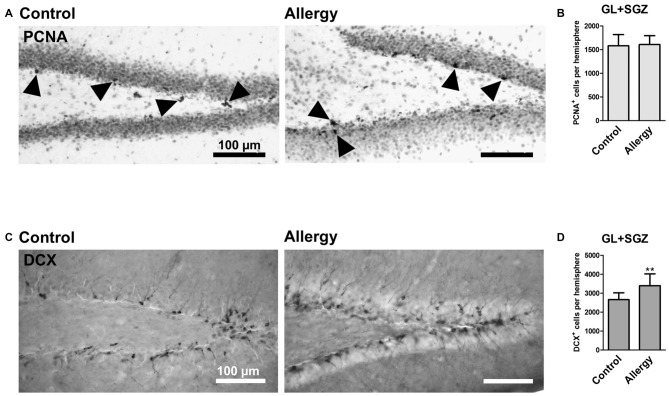
**More DCX^+^ immature neurons, but unchanged cell proliferation, in the hippocampal dentate gyrus. (A)** Proliferating cells stained with PCNA (arrowheads) in controls and the allergy group. **(B)** Allergic mice and controls had the same number of PCNA^+^ cells in GL and SGZ of the dentate gyrus. **(C)** DCX^+^ immature neurons in controls and allergy group. **(D)** Allergic mice had significantly more DCX^+^ cells in GL and SGZ. Values are depicted as Mean + SD (control: *n* = 9, allergy: *n* = 10). Statistical significance was evaluated using independent samples *t*-tests and is indicated in comparison to the control group (*p < 0.05, **p < 0.01, ***p < 0.001). Scale bars: 100 μm.

To investigate hippocampal neurogenesis in Phl p 5-sensitized mice after re-exposure to the allergen, we first quantified total cell proliferation in the GL and SGZ of the dorsal dentate gyrus. The marker PCNA labeled cells which were proliferating shortly before the animals were sacrificed, i.e., during allergen challenge (Figure 5A). This analysis showed that the number of PCNA^+^ proliferating cells in the dentate gyrus was not changed in the allergy model (control: 1579 ± 223 cells per hemisphere; allergy: 1606 ± 179 cells per hemisphere; *p* < 0.7847; Figure 5B).

Next, we evaluated if there were changes in immature DCX-expressing neurons in the hippocampal neurogenic niche (Figure 5C). Indeed, allergic mice had an increased number of DCX^+^ cells in the GL and SGZ of the dorsal dentate gyrus (control: 2659 ± 337 cells per hemisphere, allergy: 3395 ± 591 cells per hemisphere; *p* < 0.0064; Figure 5D).

Taken together these results suggest that even though the proliferation rate at the end of the experiment was not changed, either more immature neurons were generated already earlier after the sensitization phase or the differentiation of DCX^+^ cells was delayed.

### Allergy Increases Production of Mature Neurons (BrdU^+^NeuN^+^) in the Hippocampus

**Figure 6:**
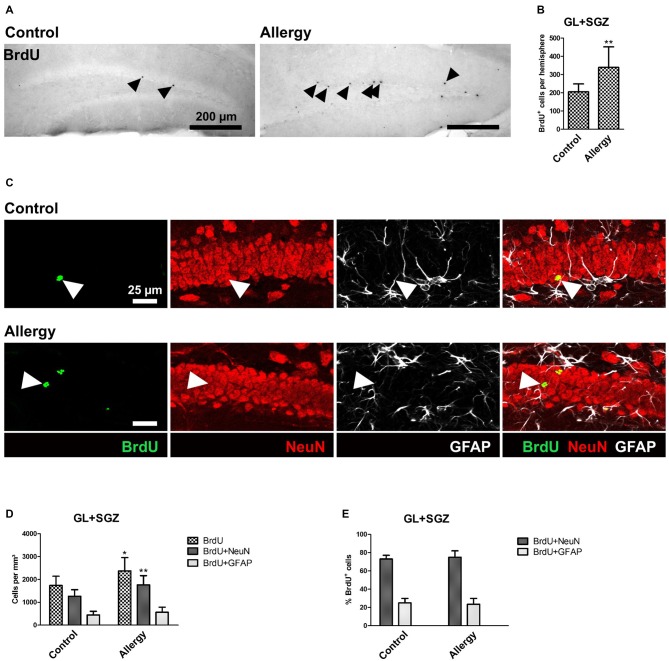
**More BrdU^+^ and BrdU^+^NeuN^+^ cells in the hippocampal dentate gyrus. (A)** BrdU^+^ cells in the dorsal dentate gyrus. BrdU was injected once 4 weeks before the end of the experiment. **(B)** Increased number of BrdU^+^ cells in the hippocampal neurogenic niche in allergic mice. **(C)** Triple labeling of BrdU (green), NeuN^+^ mature neurons (red) and GFAP^+^ astrocytes (white). BrdU^+^NeuN^+^ mature neurons are indicated by arrowheads. **(D)** In allergic mice not only the number of BrdU^+^ cells, but also of BrdU^+^NeuN^+^ mature neurons increased in comparison to controls. **(E)** The percentage of BrdU^+^ cells which became mature NeuN^+^ neurons or GFAP^+^ radial glia (or astrocytes) remained unchanged. Values are depicted as Mean + SD. (control: *n* = 9, allergy: *n* = 10). Statistical significance was evaluated using independent samples *t*-tests and is indicated in comparison to the control group (*p < 0.05, **p < 0.01, ***p < 0.001). Scale bars: **(A)** 200 μm, **(C)** 25 μm.

For cell fate analysis, mice received a single injection of BrdU after the last sensitization step and 4 weeks before the end of the experiment. BrdU, a thymidine analog, incorporates into the DNA of proliferating cells. Thus, a BrdU^+^ nucleus indicates a cell that had been dividing at the time of injection, i.e., after the sensitization was completed, and survived until the end of the experiment (Figure 6A). A quantification of the total number of BrdU^+^ cells in the GL and SGZ of the dorsal dentate gyrus showed that the allergic mice had a significantly higher number of BrdU^+^ cells than the control group (control: 206 ± 40 cells per hemisphere, allergy: 339 ± 107 cells per hemisphere; *p* < 0.0046; Figure 6B).

Since the increase in BrdU^+^ cells in the hippocampal GL and SGZ of allergic mice could not be explained by microglial cells, as their numbers actually decreased, we further analyzed the cell fate of these BrdU^+^ cells and investigated if they became NeuN^+^ mature neurons or GFAP^+^ astrocytes or radial glia (Figure 6C). In allergic mice, the number of BrdU^+^NeuN^+^ mature neurons increased significantly (control: 1264 ± 271 cells/mm^3^, allergy: 1766 ± 382 cells/mm^3^; *p* < 0.0065; Figure 6D). There was also a slight, but not significant increase in the number of BrdU^+^GFAP^+^ cells (control: 446 ± 154; 566 ± 211; *p* < 0.2027; Figure 6D). However, the percentages of BrdU^+^ cells which were either positive for NeuN or GFAP were unchanged (Figure 6E).

These results indicate that the observed increase in BrdU^+^ cells is due to an increased net production of mature NeuN^+^ neurons in the hippocampal neurogenic niche and not caused by a change in the differentiation fate of the cells.

### Allergy Neither Affects the Numbers of BrdU^+^ Cells in the SVZ nor the Cell Fate of BrdU^+^ Cells in the OB

**Figure 7:**
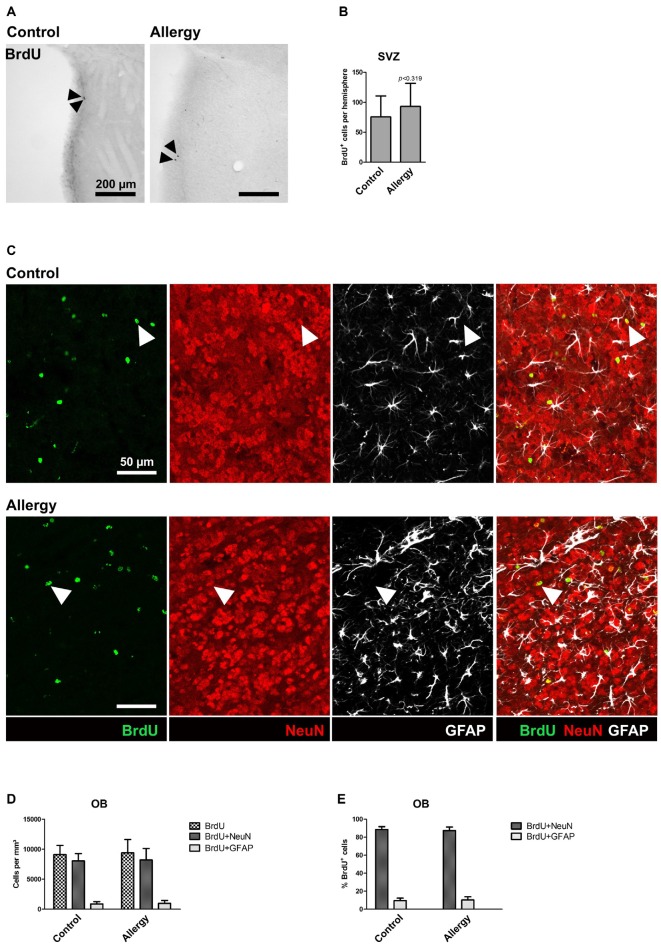
**Unchanged number of BrdU^+^ cells in the subventricular zone (SVZ) and no alterations in cell fate of BrdU^+^ cells in the OB. (A)** BrdU labeling in the SVZ. Arrowheads indicate BrdU^+^ cells. **(B)** There is no significant difference in the number of BrdU^+^ cells in the SVZ between controls and allergic mice. **(C)** BrdU labeling in the OB. The images show a triple labeling of BrdU (green) NeuN (red) and GFAP (white). Arrowheads indicate BrdU^+^NeuN^+^ cells. **(D,E)** Neither the density **(D)** nor the percentage **(E)** of BrdU^+^NeuN^+^ or BrdU^+^GFAP^+^ cells in the OB differs significantly between controls and allergic mice. Values are depicted as Mean + SD (control: *n* = 9, allergy: *n* = 10). Statistical significance was evaluated using independent samples *t*-tests and is indicated in comparison to the control group (*p < 0.05, **p < 0.01, ***p < 0.001). Scale bars: **(A)** 200 μm, **(C)** 50 μm.

To assess if the pro-neurogenic effect of allergy was specific for the hippocampus, or if it was also affecting the other classical neurogenic niche, we analyzed BrdU^+^ cells in the SVZ (Figures 7A,B) and OB (Figures 7C–E). In the SVZ, in both groups, hardly any BrdU^+^ cells were left (Figure 7A), and there was no significant difference between the groups (control: 76 ± 33 cells per hemisphere, allergy: 93 ± 37 cells per hemisphere; *p* < 0.3188; Figure 7B). Next, we did an analysis of cell fate of BrdU^+^ cells in the OB (Figures 7C–E). There was no significant difference in the densities of BrdU^+^ cells between the groups (control: 9114 ± 1425 cells/mm^3^, allergy: 9396 ± 2105 cells/mm^3^; *p* < 0.7534) and also the density of BrdU^+^NeuN^+^ (control: 8059 ± 1149 cells/mm^3^, allergy: 8216 ± 1819 cells/mm^3^; *p* < 0.8363) or BrdU^+^GFAP^+^ cells (control: 889 ± 355 cells/mm^3^, allergy: 990 ± 451 cells/mm^3^; *p* < 0.6178) did not change significantly (Figure 7D). In both cases, around 90% of these BrdU^+^ cells in the OB were also positive for NeuN (control: 88.6 ± 2.9%, allergy: 87.5 ± 3.6%; *p* < 0.4834; Figure 7E).

## Discussion

In the present study, the effects of a T_H_2-polarized systemic inflammation on the neurogenic niche in the hippocampus were analyzed in a model of grass pollen allergy. Surprisingly, allergy seems to have a positive impact on the production of new neurons and leads to a down-regulation of microglial activation in this region.

The analysis of immunological parameters in sera and lungs confirmed that in the allergy model a T_H_2-polarized allergic reaction was induced. As expected, the allergy model showed the typical immunoglobulin pattern for an allergic immune response (high levels of allergen-specific IgE and IgG1, low levels of IgG2c). In addition to a robust increase of T_H_2 cytokines in serum and BAL fluid, also a modest, but significant, induction of pro-inflammatory cytokines was observed. Moreover, CCL2 an important chemoattractant for monocytes was increased in the serum. These immune parameters are in line with what has been described for patients suffering from allergies affecting the airways (Kuna et al., 1996; Holgate et al., 1997).

Microglia are the tissue macrophages of the CNS and are responsible for CNS immune surveillance. They react to pathogenic events and are “activated” in a multi-step process (Kettenmann et al., 2011), which leads to an upregulation of specific proteins, e.g., MHCII for antigen-presentation or CD68 which is associated with lysosomes and endosomes (Boche et al., 2013). Microglia are also part of the hippocampal neurogenic niche, and have regulatory functions there (Gemma and Bachstetter, 2013; Sierra et al., 2014). The allergic immune response in our model affected these immune cells in the hippocampal neurogenic niche in an unexpected way: allergy led to a “deactivation” of microglia in this region, since both, their numbers and their MHCII expression were reduced.

Actually, there is one study showing that—in the absence of inflammatory stimuli—the rate of neurogenesis and microglial numbers, specifically in the dentate gyrus, are inversely correlated (Gebara et al., 2013). This fits to our results, since we observed an increase of neurogenesis accompanied by a reduction in microglial numbers (Iba1^+^ cells). The further reduction of microglia that are expressing MHCII (from 58.8 ± 5.1% in controls to 44.7 ± 4.6% in allergic mice, *p* < 0.00001; data not shown) might suggest that the affected microglia were either less phagocytic or less inclined to present the phagocytosed antigens as MHCII is involved in this process. A reduced phagocytic activity could be explained by a lack of cellular debris due to an increased survival of newly generated immature DCX^+^ neurons, since superfluous progenitors are normally phagocytosed by microglia (Sierra et al., 2010). However, the number of microglia expressing CD68, a widely used marker for microglial activation, which is located in phagosomes and lysosomes, was unaffected, suggesting that allergy affects predominantly antigen presentation.

At first glance, our findings are in contrast to numerous studies which found that microglia are activated by systemic inflammation (reviewed in Hoogland et al., 2015). However, those studies exclusively used stimuli inducing T_H_1-polarized immune responses, i.e., LPS, bacteria, or viruses (reviewed in Hoogland et al., 2015), which could explain the different outcome. Moreover, in our allergy model, the deactivation of microglia seemed to be specific for the hippocampal dentate gyrus, since we actually observed an activation of microglia accompanied by upregulation of MHCII and CD68 in the OB (Figure 4). The OB is not only the region into which SVZ-derived newly generated neurons are integrated, but also seems to contain an especially reactive sub-population of microglia (Lalancette-Hebert et al., 2009), which could explain why allergy has an opposite effect on microglia in this region. Alternatively, the intranasal delivery of the allergen might have a much more pronounced effect on the OB system compared to the hippocampus. For this reason, it might be interesting to investigate whether this difference between hippocampal and OB microglia is also present in allergy models affecting other parts of the organism, e.g., the skin.

Why are microglia deactivated in the hippocampus of allergic mice? It is tempting to assume that this might be a regulatory mechanism protecting the hippocampus, which is central for many important processes, from the immune response in the periphery. An alternative hypothesis would be that this down-regulation is directly caused by the elevated levels of T_H_2 cytokines in the blood. It is even more challenging to speculate about the functional consequences of this observed down-regulation of microglial activation below the normal “surveying state” in the young hippocampus. If immune surveillance in the hippocampus is down-regulated for extended periods, this may have detrimental consequences. However, with the current experimental set-up we do not know if this microglial deactivation is transient or persists for longer periods. Of course, the allergy-induced changes in hippocampal microglia were rather subtle, which could be due to the fact that the starting point for the down-regulation was a young healthy condition. Therefore, it would be highly interesting to investigate what allergy does to microglia in the aged CNS, which might already be somewhat primed for a pro-inflammatory activation (Norden and Godbout, 2013).

Concomitantly with microglial deactivation, hippocampal neurogenesis was increased, i.e., we observed higher numbers of DCX^+^ immature neurons and BrdU^+^NeuN^+^ mature neurons. Since BrdU was injected after the sensitization period (4 weeks before the end of the experiment) and the allergen challenge started only 4 days before the animals were sacrificed, it seems that already the sensitization has an impact on hippocampal neurogenesis. With the current experimental setup it is only possible to analyze the cumulative effect of both phases, but it would be interesting to study the effect of sensitization alone, and further time points in which also the challenge period is extended. Additionally, it would be worth investigating whether alternative sensitization and challenge routes (e.g., in a model for food allergy) have a similar impact on microglia and neurogenesis in the hippocampus.

So far, we can only hypothesize that the observed increase in hippocampal neurogenesis may also have functional consequences on long-term potentiation or learning and memory. For this, further studies including electrophysiological analysis and behavioral tests are needed.

We did not observe any changes in proliferation at the end of the challenge period. This is in contrast to a study which showed that in immature mice, chronic asthma leads to a reduced proliferation in the hippocampal neurogenic niche (Guo et al., 2013). An explanation for these different results could be that the latter study particularly investigated animals of a very young and thus, probably more vulnerable age, starting the OVA sensitization in 3-weeks-old mice and then extended the OVA challenge over a period of 9 weeks (Guo et al., 2013), whereas the present allergy model started in young adult mice (2-months-old) and the actual challenge period only lasted for 4 days. Moreover, commercially available OVA often contains substantial levels of LPS, which lead to additional and potentially confounding immune responses, whereas the Phl p 5 used in the present study was essentially LPS-free. However, similar to the study of Guo et al. (2013), also in our model an increase of serum levels of VEGFα was observed. Within the neurogenic niche, VEGFα supports neurogenesis (Schänzer et al., 2004; reviewed in Kokaia et al., 2012), and it also has been shown that peripheral VEGF is necessary for exercise-induced neurogenesis (Fabel et al., 2003). Taken together, these results suggest that a peripheral increase of VEGF might affect neurogenesis, but this is probably context-dependent.

High serum levels of CCL11 (eotaxin-1) were recently described to inhibit hippocampal neurogenesis (Villeda et al., 2011). While in the BAL fluid, as expected, elevated levels of CCL11 were only found in the allergy group, in serum both, control mice as well as allergic mice, displayed high levels of CCL11. In fact, serum levels of CCL11 were even slightly decreased in allergic mice. This is in line with data showing that eotaxin is elevated in nasal secretion of allergic patients during the pollen season, whereas no difference was observed in eotaxin serum levels—neither in or out of pollen season, nor between healthy and allergic donors (Pullerits et al., 2000).

Depending on the type of immune response, systemic inflammation might elicit diverse effects on the CNS. Our data suggests that a T_H_2-polarized allergic immune response might promote neurogenesis and down-regulate microglia in the hippocampus. However, at the moment it is not clear if this also has a beneficial effect on CNS functions and what happens if this immune response persists for a longer time. Clearly, more experiments investigating the impact of different types of systemic inflammation on the CNS are needed to further the understanding about the interplay between peripheral immune activation and CNS functions.

## Author Contributions

BK: designed the study, analyzed and interpreted data, did histology and microscopy, and drafted the manuscript. HM: participated in the histology. SS participated in the analysis of the immunological parameters in sera and lung fluids. JT: participated in the design of the study and discussion of the results. RW: designed the study, analyzed and interpreted data, carried out the animal treatments and analysis of the blood and lung parameters. LA: participated in the study design and coordination, and the discussion of the results. All authors contributed to revising the manuscript and approved the final version.

## Funding

The research leading to these results has received funding from the European Union’s Seventh Framework Programme (FP7/2007-2013) under grant agreement number HEALTH-F2-2011-278850 (INMiND) and by the Research Fund of the Paracelsus Medical University Salzburg (PMU-FFF) as a RISE Project under grant agreement R-13/02/046-KLE, and the Allergy-Cancer-BioNano Research Center of the University of Salzburg.

## Conflict of Interest Statement

The authors declare that the research was conducted in the absence of any commercial or financial relationships that could be construed as a potential conflict of interest.

## Footnotes

^1^http://graphpad.com/quickcalcs/grubbs1.
